# Characteristics and Outcomes of Patients With Pregnancy-Related End-Stage Kidney Disease

**DOI:** 10.1001/jamanetworkopen.2023.46314

**Published:** 2023-12-08

**Authors:** Lauren M. Kucirka, Ana M. Angarita, Tracy A. Manuck, Kim A. Boggess, Vimal K. Derebail, Mollie E. Wood, Michelle L. Meyer, Dorry L. Segev, Monica L. Reynolds

**Affiliations:** 1 Division of Maternal Fetal Medicine, Department of Obstetrics and Gynecology, University of North Carolina at Chapel Hill; 2 Division of Maternal Fetal Medicine, Department of Obstetrics and Gynecology, Sidney Kimmel Medical College of Thomas Jefferson University, Philadelphia, Pennsylvania; 3 Institute for Environmental Health Solutions, Gillings School of Global Public Health, Chapel Hill, North Carolina; 4 UNC Kidney Center, Division of Nephrology and Hypertension, Department of Medicine, University of North Carolina at Chapel Hill; 5 Department of Epidemiology, Gillings School of Public Health, University of North Carolina at Chapel Hill; 6 Center for Pharmacoepidemiology, University of North Carolina at Chapel Hill; 7 Department of Emergency Medicine, University of North Carolina at Chapel Hill; 8 Division of Transplant, Department of Surgery, New York University Langone Medical Center, New York

## Abstract

**Importance:**

The incidence of pregnancy-related acute kidney injury is increasing and is associated with significant maternal morbidity including progression to end-stage kidney disease (ESKD). Little is known about characteristics and long-term outcomes of patients who develop pregnancy-related ESKD.

**Objectives:**

To examine the characteristics and clinical outcomes of patients with pregnancy-related ESKD and to investigate associations between pre-ESKD nephrology care and outcomes.

**Design, Setting, and Participants:**

This was a cohort study of 183 640 reproductive-aged women with incident ESKD between January 1, 2000, and November 20, 2020, from the US Renal Data System and maternal data from births captured in the US Centers for Disease Control and Prevention publicly available natality data. Data were analyzed from December 2022 to June 2023.

**Exposure:**

Pregnancy-related primary cause of ESKD, per *International Classification of Diseases, Ninth Revision (ICD-9)* and *ICD-10* codes reported at ESKD onset by the primary nephrologist on Centers for Medicare and Medicaid Services form 2728.

**Main Outcomes Measures:**

Multivariable Cox proportional hazards and competing risk models were constructed to examine time to (1) mortality, (2) access to kidney transplant (joining the waiting list or receiving a live donor transplant), and (3) receipt of transplant after joining the waitlist.

**Results:**

A total of 341 patients with a pregnancy-related primary cause of ESKD were identified (mean [SD] age 30.2 [7.3]). Compared with the general US birthing population, Black patients were overrepresented among those with pregnancy-related ESKD (109 patients [31.9%] vs 585 268 patients [16.2%]). In adjusted analyses, patients with pregnancy-related ESKD had similar or lower hazards of mortality compared with those with glomerulonephritis or cystic kidney disease (adjusted hazard ratio [aHR], 0.96; 95% CI, 0.76-1.19), diabetes or hypertension (aHR, 0.49; 95% CI, 0.39-0.61), or other or unknown primary causes of ESKD (aHR, 0.60; 95% CI, 0.48-0.75). Despite this, patients with pregnancy-related ESKD had significantly lower access to kidney transplant compared with those with other causes of ESKD, including (1) glomerulonephritis or cystic kidney disease (adjusted subhazard ratio [aSHR], 0.51; 95% CI, 0.43-0.66), (2) diabetes or hypertension (aSHR, 0.81; 95% CI, 0.67-0.98), and (3) other or unkown cause (aSHR, 0.82; 95% CI, 0.67-0.99). Those with pregnancy-related ESKD were less likely to have nephrology care or have a graft or arteriovenous fistula placed before ESKD onset (nephrology care: adjusted relative risk [aRR], 0.47; 95% CI, 0.40-0.56; graft or arteriovenous fistula placed: aRR, 0.31; 95% CI, 0.17-0.57).

**Conclusion and Relevance:**

In this study, those with pregnancy-related ESKD had reduced access to transplant and nephrology care, which could exacerbate existing disparities in a disproportionately Black population. Increased access to care could improve quality of life and health outcomes among these young adults with high potential for long-term survival.

## Key Points

**Question:**

What are the long-term outcomes of patients with pregnancy-related end-stage kidney disease (ESKD)?

**Findings:**

This cohort study of 183 640 women found that Black patients were overrepresented among those with pregnancy-related ESKD compared with the general birthing population (31.9% vs 16.2%). Compared with reproductive age patients with other causes of ESKD, those with pregnancy-related ESKD were significantly less likely to have access to kidney transplant or nephrology care before ESKD onset, despite having equivalent or better survival with ESKD.

**Meaning:**

These findings suggest there are significant disparities in access to kidney transplant and nephrology care in this disproportionately Black population; improving postpartum care should be a priority.

## Teaser

This cohort study evaluates characteristics and clinical outcomes of patients with pregnancy-related end-stage kidney disease.

## Introduction

Compared with other developed nations, US rates of maternal mortality and severe maternal morbidity are significantly higher.^1,2,3^ Acute kidney injury (AKI) during pregnancy is a major cause of morbidity with a high risk of adverse outcomes including 13-fold higher odds of maternal mortality, 9-fold higher odds of cardiovascular events, and a 30% to 60% risk of fetal mortality and morbidity.^4,5,6^ In addition, there are stark disparities in pregnancy outcomes, with Black patients 3 times more likely than White patients to die from a pregnancy-related cause.^7,8,9,10^ Black patients appear to be disproportionately affected; for example, Michigan’s severe maternal morbidity review committee reported 32.6 cases of AKI per 10 000 hospitalizations for Black patients vs 10.8 for White patients.^11^ While the reasons for this are likely multifactorial, structural racism may serve as a root cause of inequities in maternal health outcomes.^12,13^ Although it was previously decreasing in developed countries due to improvements in prenatal care and fewer unsafe abortions, pregnancy-associated AKI has been on the rise in recent decades, with incidence more than doubling from 2.4 to 6.3 cases per 10 000 between 1999 and 2011.^14,15,16^ In light of the recent Supreme Court decision in *Dobbs v. Jackson* further restricting abortion access, rates of pregnancy-related kidney disease will likely continue to increase.^17,18,19^

In its most extreme form, AKI can result in end-stage kidney disease (ESKD) requiring dialysis or kidney transplant. A recent systematic review and meta-analysis^20^ including 845 pregnancies affected by AKI estimated that pregnancy-related AKI resulted in ESKD in 2.4% of cases. This estimation is likely conservative as it may not capture ESKD that does not occur immediately but develops in the months to years following a pregnancy impacted by AKI. Little is known about the characteristics and long-term outcomes of those who develop pregnancy-related ESKD. Understanding the factors associated with risk and clinical course of pregnancy-related ESKD is critical to both inform counseling for this vulnerable population and to develop strategies to optimize outcomes. Our objectives were to (1) describe the demographic and clinical characteristics of patients who developed pregnancy-related ESKD in a national cohort; (2) examine ESKD outcomes, including survival and access to kidney transplantation, for those with pregnancy-related ESKD compared with reproductive age women with other causes of ESKD; and (3) examine differences in predialysis care for patients with pregnancy-related ESKD.

## Methods

### Study Design

Data were drawn from the US Renal Data System (USRDS), a national registry of all patients with ESKD in the US,^21^ and publicly available birth certificate data from the US Centers for Disease Control and Prevention (CDC) National Center for Health Statistics.^22^ The USRDS is linked to the Social Security Master Death File and the United Network for Organ Sharing (UNOS), providing data on long-term transplant outcomes and mortality. Our study population included 183 640 persons of reproductive age (14-50 years) who initiated dialysis or received a preemptive kidney transplant between January 1, 2000, and November 20, 2020; were identified as female sex; and had a primary cause of kidney failure recorded per the Centers for Medicare and Medicaid Services (CMS) Medical Evidence form 2728. We excluded 87 patients who were missing form 2728 and 2586 patients who were missing primary cause of kidney failure. This form is required to be filled out by the supervising physician within 45 days of dialysis initiation or kidney transplant receipt. Although our primary analyses used data drawn from USRDS, we compared the demographic and clinical characteristics of patients with pregnancy-related kidney failure with the general population of birthing patients in the US as captured in CDC birth certificate data in the years 2000 and 2020.

This study was reviewed by the institutional review board at the University of North Carolina (UNC) School of Medicine and qualified for an exemption from informed consent under the Code of Federal Regulations, Protection of Human Subjects (45 CFR 46 101b) as study participants were not identifiable. A data use agreement between UNC and the National Institute of Diabetes and Digestive and Kidney Diseases through the USRDS Coordinating Center was in place. We followed the Strengthening the Reporting of Observational Studies in Epidemiology (STROBE) reporting guideline for observational studies.^23^

### Primary Cause of Kidney Failure

Primary cause of kidney failure was ascertained via CMS form 2728. A pregnancy-related primary cause of ESKD was identified if any of the following *ICD-9* or *ICD-10* diagnosis codes were reported per form 2728: 64620, 64620A, 64620a, 64620Z, 64620z, 6462Z, 6462z, or O904. Although pregnancy may have been a contributing cause in some cases, our study only captures patients in which pregnancy was considered the primary cause of ESKD. Those with a nonpregnancy-related primary cause of kidney failure were categorized as follows: (1) diabetes, (2) hypertension, (3) glomerulonephritis, (4) cystic kidney disease, or (5) other or unknown cause. For the purposes of analysis, we grouped those with diabetes or hypertension as well as those with glomerulonephritis or cystic kidney disease together as baseline characteristics were similar.

### Baseline Characteristics

Using publicly available birth certificate data for all live births from the CDC Division of Vital Statistics,^22^ demographic and clinical characteristics were compared between patients with pregnancy-related ESKD and the general population of pregnant patients during the same period. We show CDC data from the years 2000 and 2020. For this comparison, we were limited to characteristics captured both in USRDS and in the birth certificate data (age, race, ethnicity, body mass index, smoking status, diabetes, and chronic hypertension). Drawing solely from USRDS data, we separately compared demographic and clinical characteristics between those with pregnancy-related ESKD and patients with other causes of ESKD using the categories previously described. Race and ethnicity were derived as reported on CMS form 2728, which is reviewed and signed by both the reporting physician and the patient. Race and ethnicity were assessed in this study to evaluate racial disparities in maternal outcomes that have been previously identified.

### Survival and Transplant Outcomes in Patients With Pregnancy-Related ESKD

Patients were followed up from ESKD onset until death or end of study (November 20, 2020). Death was ascertained via linkage to the Social Security Master Death File.^21^ Transplant outcomes were ascertained via linkage to the UNOS standard analysis files. Access to kidney transplant was defined as either joining the deceased donor waiting list or receiving a kidney transplant from a living donor. We used the Kaplan-Meier method and the log-rank test to compare survival, access to kidney transplant, and time to transplant after joining the waitlist between those with pregnancy-related ESKD and ESKD from other causes.

Multivariable Cox proportional hazards models were built to compare the hazard of death with ESKD by primary cause of ESKD. Differences by cause of ESKD in (1) access to kidney transplant and (2) time to kidney transplant after joining the waitlist (among the subgroup who joined the deceased donor waitlist) were assessed using competing risk models per the methods of Fine and Gray,^24^ treating death as a competing risk. All models were adjusted for age, race, ethnicity, insurance type (categorized as private insurance, Medicare or both Medicare and Medicaid, Medicare only, other insurance, or no insurance), currently employed, and the presence or absence of comorbidities, including congestive heart failure, chronic obstructive pulmonary disease, cerebrovascular disease, hypertension, alcohol abuse, current smoker, and inability to ambulate. All comorbidities were included as binary variables where 0 = absent and 1 = present. For all models, the proportional hazards assumption was tested according to scaled Schoenfeld residuals.

In all statistical models, patients with a pregnancy-related primary cause of ESKD were used as the reference group. Since we were primarily interested in understanding outcomes among those with pregnancy-related ESKD, inverse hazard ratios were reported for cause of kidney failure in all models to facilitate interpretability. In other words, the reported hazard ratio for each cause of kidney failure represents the hazard of the outcome for patients with pregnancy-related ESKD as compared with patients with that cause, with a hazard ratio greater than 1 indicating an increased risk for the pregnancy-related ESKD group.

### Differences in Pre-ESKD Care

To explore differences in pre-ESKD care, we examined whether patients had (1) access to nephrology care, (2) arteriovenous graft or fistula, or (3) been informed about the option of kidney transplant before ESKD onset, as reported by the nephrologist on form 2728. These analyses were restricted to those with ESKD onset after 2005 since this information was not captured on prior versions of form 2728. We built multivariable modified Poisson regression models per the methods of Zou et al^25^ to examine the association between cause of ESKD and each of the above outcomes, adjusted for clinical and demographic factors as detailed previously. To explore the hypothesis that access to pre-ESKD care might attenuate the observed disparities for patients with pregnancy-related ESKD, we rebuilt the competing risk models with the outcome of access to transplant as follows: (1) stratified by the presence or absence of each of the above factors, and (2) including interaction terms for each factor with pregnancy-related cause of kidney failure.

### Sensitivity Analysis: Survival in an Age-Matched Cohort

Given the younger age distribution among those with pregnancy-related ESKD as compared with other causes and the potential for age to confound the relationship between ESKD cause and mortality, all analyses with death as the outcome of interest were repeated in an age-matched cohort. Specifically, patients with pregnancy-related ESKD were matched on age within a 1-year radius to patients with other causes of ESKD. Five age-matched controls were selected for each patient with pregnancy-related ESKD. The previously described Cox proportional hazards model with death as the outcome was rebuilt in the age-matched cohort.

### Statistical Analysis

All analyses were performed using multiprocessor Stata version 17.0/MP for Linux (StataCorp). *P* values less than .05 were considered statistically significant. All hypothesis tests were 2-sided and included *t* tests for continuous variables and χ^2^ tests for categorical variables. Data were analyzed from December 2022 to June 2023.

## Results

### Demographic and Clinical Characteristics, Pregnancy-Related ESKD Compared With the General US Birthing Population (per CDC)

A total of 341 patients with a pregnancy-related primary cause of ESKD were identified. Compared with the US general birthing population over the same period (per CDC), those who developed pregnancy-related ESKD were similar in age (mean [SD] 30.2 [7.3] vs 27.2 [6.2] in 2000 and 29.2 [5.8] in 2020) (Table 1). Individuals identified as Black race were overrepresented among those with pregnancy-related ESKD (109 patients [31.9%] vs 622 743 patients [15.3%] in 2000 and 585 268 [16.2%] in 2020), and White individuals were underrepresented vs the general birthing population (196 patients [57.5%] vs 3 198 673 patients [78.7%] in 2000 and 2 652 967 patients [73.3%] in 2020). The percentages of patients identified as Hispanic ethnicity were similar (78 patients [22.9%] vs 863 822 patients [21.3%] in 2000 and 905 602 patients [25.0%] in 2020). Diabetes was present in 5.3% of patients (18 patients) with pregnancy-related ESKD, compared with only 1.1% of the general birthing population in 2020 (38 105 patients). Chronic hypertension was present in 68.8% of those with pregnancy-related ESKD (201 patients) vs 0.7% of the general birthing population in 2000 (30 265 patients) and 2.5% in 2020 (91 568 patients).

**Table 1.** Demographic and Clinical Characteristics of Patients With Pregnancy-Related End-Stage Kidney Disease (ESKD) Compared With the General Birthing Population as Captured in Publicly Available Natality Data From US Centers for Disease Control and Prevention

Characteristic	Participants, No. (%)		
	Pregnancy-related ESKD (n = 341)	General birthing population in 2000 (n = 4 063 823)^a^	General birthing population in 2020 (n = 3 619 826)^a^
Age, mean (SD), y	30.2 (7.3)	27.2 (6.2)	29.2 (5.8)
Age, median (IQR)	30.0 (25.0-35.0)	27.0 (22.0-32.0)	29.0 (25-33)
Race			
Asian or Pacific Islander	19 (5.6)	200 722 (4.9)	244 869 (6.8)
Black	109 (31.9)	622 743 (15.3)	585 268 (16.2)
White	196 (57.5)	3 198 673 (78.7)	2 652 967 (73.3)
Other or unknown^b^	17 (5.0)	41 685 (1.0)	136 722 (3.8)
Hispanic ethnicity	78 (22.9)	863 822 (21.3)	905 602 (25.0)
Smoker^c^	16 (5.5)	425 102 (10.7)	269 433 (7.4)
Body mass index, mean (SD)^d^	27.1 (7.2)	NR	28.9 (12.1)
Body mass index, median (IQR)^d^	27.4 (22.7-33.7)	NR	26.3 (22.6-31.6)
Diabetes	18 (5.3)	117 289 (2.9)^e^	38 105 (1.1)
Hypertension^c^	201 (68.8)	30 265 (0.7)	91 568 (2.5)

Abbreviation: NR, not reported.

^a^ From US Centers for Disease Control and Prevention natality data.

^b^ Other includes American Indian/Alaska Native and those who identified as other race.

^c^ Denominator for column 1 is 292.

^d^ Body mass index is calculated as weight in kilograms divided by height in meters squared.

^e^ Data reported as diabetes only (no distinction between pregestational and gestational in 2000 data set; pregestational diabetes is specified in 2020 data set and this is what is reported in table).

### Demographic and Clinical Characteristics, Pregnancy-Related ESKD Compared With Other Causes of ESKD in Reproductive Age Women (per USRDS)

Of 183 640 patients with ESKD aged 14 to 50 who were identified as female, 341 (0.19%) had a pregnancy-related primary cause of ESKD per CMS form 2728 (Table 2). Nonpregnancy-related causes of ESKD included diabetes (69 935 patients [38.1%]), hypertension (36 433 patients [19.8%]), glomerulonephritis (40 379 patients [21.9%]), cystic kidney disease (8492 patients [4.6%]), or other or unknown cause (28 060 patients [15.3%]). Compared with patients with other primary causes of ESKD, patients with pregnancy-related ESKD were younger, more likely to report Hispanic ethnicity, have Medicaid only for insurance, and be currently employed or in school. For patients with pregnancy-related ESKD, rates of comorbidities such as congestive heart failure, chronic obstructive pulmonary disease, and cerebrovascular disease were similar to patients with cystic kidney disease or glomerulonephritis and lower than those with diabetes or hypertension as the primary cause of ESKD. Although Black patients with pregnancy-related ESKD were overrepresented when compared with the general population of pregnancies resulting in live births in the US, there were fewer differences by cause of ESKD, with Black patients comprising 32.0% (109 patients), 33.1% (16 193 patients), 43.9% (46 656 patients), and 32.0% (8983 patients) of those with pregnancy-related, cystic kidney disease or glomerulonephritis, diabetes or hypertension, and other or unknown causes, respectively.

**Table 2.** Demographic and Clinical Characteristics and Outcomes of Reproductive-Age Female Patients by Cause of End-Stage Kidney Disease

Characteristic	No. (%)			
	Pregnancy-related (n = 341)	Glomerulonephritis/cystic kidney (n = 48 871)	Diabetes/hypertension (n = 106 368)	Other/unknown (n = 28 060)
Demographics				
Age, mean (SD), y	30.2 (7.3)	36.1 (9.8)	41.4 (7.3)	37.6 (9.9)
Race				
Asian or Pacific Islander	19 (5.6)	3710 (7.6)	4841 (4.6)	1148 (4.1)
Black	109 (32.0)	16 193 (33.1)	46 656 (43.9)	8983 (32.0)
White	196 (57.5)	27 656 (56.6)	51 699 (48.6)	17 264 (61.5)
Other or unknown^a^	17 (5.0)	1312 (2.7)	3172 (3.0)	665 (2.4)
Hispanic ethnicity	78 (22.9)	9305 (19.1)	19 689 (18.5)	4006 (14.6)
Insurance type				
Private insurance	95 (27.9)	20 291 (41.6)	26 859 (25.3)	8499 (30.9)
Medicare or both Medicare and Medicaid	16 (4.7)	5207 (10.6)	19 406 (18.2)	4056 (14.7)
Medicaid only	150 (43.9)	12 502 (25.6)	35 230 (33.2)	8681 (31.5)
Other insurance	35 (10.2)	5251 (10.6)	9121 (8.6)	2769 (10.1)
No insurance	45 (13.2)	5568 (11.4)	15 637 (14.7)	3531 (12.8)
Currently employed or in school	264 (77.4)	35 037 (71.8)	74 645 (70.2)	18 521 (67.2)
Clinical characteristics				
Body mass index (SD)^b^	27.1 (7.2)	28.4 (8.6)	31.6 (9.5)	27.7 (9.0)
CHF, COPD, CVD, or inability to ambulate^c^	37 (12.7)	5116 (11.1)	28 333 (27.3)	4587 (18.5)
Hypertension^c^	201 (68.8)	39 255 (85.5)	39 355 (90.8)	16 160 (65.2)
Outcomes				
Died	85 (24.9)	35 268 (27.8)	50 517 (52.5)	15 589 (44.4)
Access to transplant	141 (41.4)	31 526 (64.5)	36 430 (34.3)	11 921 (42.5)

Abbreviations: CHF, congestive heart failure; COPD, chronic obstructive pulmonary disease; CVD, cerebrovascular disease.

^a^ Other includes American Indian/Alaska Native and those who identified as other race.

^b^ Body mass index is calculated as weight in kilograms divided by height in meters squared.

^c^ Denominators 292, 45 900, 103 891, 24 789 for columns 1 to 4, respectively.

### Survival in Full and Age-Matched Cohort

Survival was lowest among those with diabetes or hypertension as the primary cause of ESKD, followed by those with other or unknown cause (Figure, A). ESKD survival was highest among those with pregnancy-related causes and glomerulonephritis or cystic kidney disease. Results were similar in a cohort matched on age within a 1-year radius (Figure, B). In a multivariable Cox proportional hazards model, those with pregnancy-related ESKD had a similar hazard of death compared with patients with glomerulonephritis or cystic kidney disease (adjusted inverse hazard ratio [aHR], 0.96; 95% CI, 0.76-1.19; *P* = .79) (Table 3), and lower hazards compared with those with diabetes or hypertension (aHR, 0.49; 95% CI, 0.39-0.61) or other or unknown causes (aHR, 0.60; 95% CI, 0.48-0.75). Full details of this model can be found in eTable 1 in Supplement 1. Inferences were similar when the Cox proportional hazards model was built using the age-matched cohort (see eTable 2 in Supplement 1 for full model results).

**Figure.**
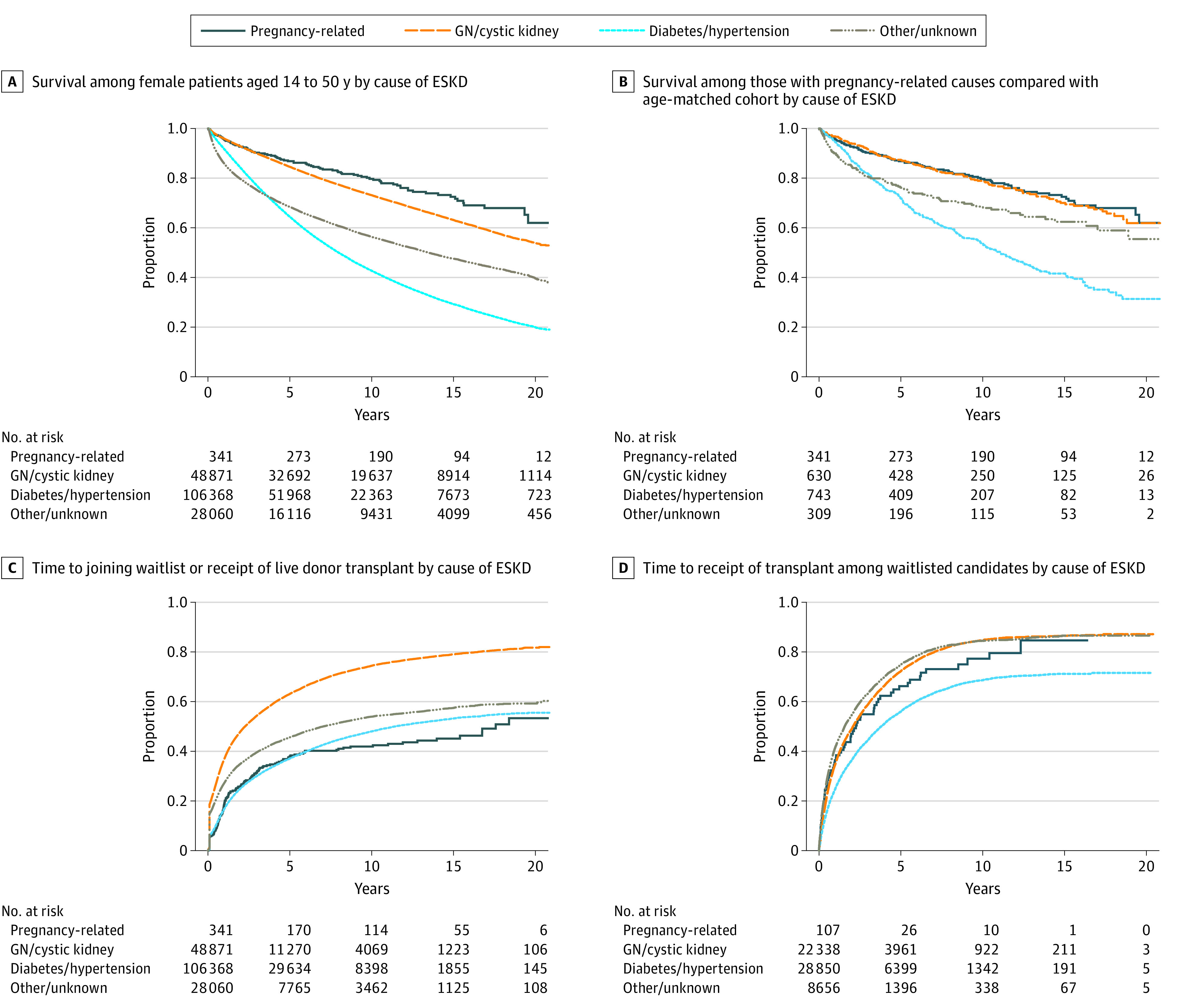
Survival Among Those With Pregnancy-Related End-Stage Kidney Disease (ESKD) Compared With Those With Other Causes GN indicates glomerulonephritis.

**Table 3.** Multivariable Cox Proportional Hazards Model: Time to Death and Multivariable Competing Risk Models for Time to Access to Transplant (Joining Kidney Transplant Waitlist or Receipt of Live Donor Transplant), and Time to Transplant After Joining Waitlist, Among Reproductive-Age Patients With End-Stage Kidney Disease, by Primary Cause of Kidney Failure^a^

Models	aHR (95% CI)^b^	*P* value
Death		
Pregnancy-related vs	1 [Reference]	NA
Glomerulonephritis/cystic kidney	0.96 (0.76-1.19)	.79
Diabetes/hypertension	0.49 (0.39-0.61)	<.001
Other/unknown	0.60 (0.48-0.75)	<.001
Access to transplant, aSHR (95% CI)^b^		
Pregnancy-related vs	1 [Reference]	NA
Glomerulonephritis/cystic kidney	0.51 (0.43-0.63)	<.001
Diabetes/hypertension	0.81 (0.67-0.98)	.02
Other/unknown	0.82 (0.67-0.99)	.03
Time to transplant after joining waitlist, aSHR (95% CI)^b^		
Pregnancy-related vs	1 [Reference]	NA
Glomerulonephritis/cystic kidney	0.91 (0.72-1.16)	.44
Diabetes/hypertension	1.23 (0.95-1.54)	.11
Other/unknown	0.92 (0.71-1.16)	.45

Abbreviations: aHR, adjusted hazard ratio; aSHR, adjusted subhazard ratio; NA, not applicable.

^a^ Inverse hazard ratios shown for cause of kidney failure; these should be interpreted as the hazard for patients with pregnancy-related end-stage kidney disease compared with each of the other causes.

^b^ Adjusted for potential confounders, including race, Hispanic ethnicity, insurance type, current employment, and comorbidities, including congestive heart failure, chronic obstructive pulmonary disease, cerebrovascular disease, alcohol dependence, hypertension, inability to ambulate, and current smoker.

### Access to Kidney Transplant

Patients with pregnancy-related ESKD were less likely to have access to kidney transplant than those with glomerulonephritis or cystic kidney disease, with transplant rates similar to those with diabetes or hypertension as the primary cause (Figure, C). Disparities in access to transplant for patients with pregnancy-related ESKD were attenuated when the analysis was restricted only to those who joined the deceased donor waiting list (Figure, D). These findings persisted even after adjustment for demographic and clinical characteristics; patients with pregnancy-related ESKD had reduced access to transplant compared with those with glomerulonephritis or cystic kidney disease (adjusted inverse subhazard ratio, [aSHR], 0.51; 95% CI, 0.43-0.63), diabetes or hypertension (aSHR, 0.81; 95% CI, 0.67-0.98), or other or unknown cause (aSHR, 0.82; 95% CI, 0.67-0.99) (Table 3). In contrast, when the analysis was restricted to those who joined the deceased donor waitlist, there was not a statistically significant difference in time to receipt of kidney transplant between pregnancy-related ESKD and other causes (Table 3). Full details of this model are available in eTable 3 in Supplement 1.

### Access to Predialysis Care

Compared with their counterparts with other causes of ESKD, patients with pregnancy-related ESKD were less likely to have access to nephrology care before ESKD onset (33.6% vs 58.1%-77.6%) or have a graft or fistula placed before ESKD onset (4.4% vs 11.3%-18.0%); these differences persisted in models adjusted for demographic and clinical characteristics (Table 4). They were less likely to be informed about the option of kidney transplant compared with those with glomerulonephritis or cystic kidney or diabetes or hypertension (84.4% vs 88.7%-91.7%), and were informed at similar rates to those with other or unknown causes (84.4% vs 83.6%). Those with pregnancy-related ESKD were less likely to have nephrology care or have a graft or arteriovenous fistula placed before ESKD onset (nephrology care: adjusted relative risk [aRR], 0.47; 95% CI, 0.40-0.56; graft or arteriovenous fistula placed: aRR, 0.31; 95% CI, 0.17-0.57). In stratified analyses, disparities in access to transplant for patients with pregnancy-related ESKD appeared attenuated in those who had early access to nephrology care or had a graft or fistula placed before ESKD onset; however, interaction terms for each factor with pregnancy-related ESKD were not statistically significant (eTable 4 in Supplement 1).

**Table 4.** Multivariable Modified Poisson Regression Models: Association Between Cause of Kidney Failure and Access To Care^a^

Cause of ESKD	Early access to nephrology care		Graft or arteriovenous fistula at ESKD onset		Patient informed about option of transplant	
	%	aRR (95% CI)^b^	%	aRR (95% CI)^b^	%	aRR (95% CI)
Pregnancy-related vs	33.6	1 [Reference]	4.4	1 [Reference]	84.4	1 [Reference]
GN/cystic kidney	77.6	0.47 (0.40-0.56)	18.0	0.31 (0.17-0.57)	91.7	0.93 (0.87-0.97)
Hypertension/diabetes	68.1	0.52 (0.44-0.62)	14.8	0.40 (0.22-0.74)	88.7	0.93 (0.88-0.99)
Other/unknown	58.1	0.60 (0.51-0.72)	11.3	0.47 (0.26-0.86)	83.6	0.99 (0.93-1.04)

Abbreviations: aRR, adjusted relative risk; ESKD, end-stage kidney disease; GN, glomerulonephritis.

^a^ Inverse relative risk and hazard ratios shown for cause of kidney failure; these should be interpreted as the hazard/relative risk for patients with pregnancy-related ESKD compared with other causes.

^b^ Each model adjusted for age, race, ethnicity, insurance type, currently employed, and the presence or absence of comorbidities, including congestive heart failure, chronic obstructive pulmonary disease, cerebrovascular disease, hypertension, alcohol abuse, current smoker, and inability to ambulate.

## Discussion

Pregnancy-associated AKI is a major cause of maternal mortality and severe morbidity, including progression to ESKD.^4,20,26,27,28^ Our data show that Black patients are at highest risk for pregnancy-related ESKD, comprising nearly one-third of these individuals compared with only 16.2% of the general population giving birth in the US. Although survival was equivalent or better for patients with pregnancy-related ESKD compared with similar patients with other causes, we observed stark disparities in access to transplant and nephrology care, with patients half as likely to have nephrology care or have a graft or fistula placed before ESKD onset compared with those with other causes of ESKD. Notably, we found that receipt of predialysis care lessened the observed disparities in access to transplant, highlighting that early comprehensive care to this population deserves further dedicated efforts.

To our knowledge, this is the largest study of patients with a pregnancy-related primary cause of ESKD, and the first to assess outcomes as compared with those with nonpregnancy-related causes. Prior studies have shown that 2.4% (95% CI, 1.3%-4.2%) of pregnancy-related AKI cases result in ESKD.^20^ We found that only a small percentage of individuals (341 patients [0.19%]) had pregnancy-related ESKD per CMS form 2728; however, this likely underestimates the true burden because our study only includes patients whose primary cause of ESKD was felt to be pregnancy related, and does not account for those who had pregnancy as a secondary contributing cause of ESKD, perhaps in the months to years following a pregnancy-induced kidney injury. In the US, over the past decade, the incidence of pregnancy-related AKI has nearly tripled after initially declining due to improvements in obstetric care and fewer unsafe abortions.^16^ However, septic abortion is the most common cause of pregnancy-related AKI in developing nations and may comprise up to 25% of dialysis center referrals in that setting.^29,30,31^ In light of the Supreme Court decision in *Dobbs v. Jackson* further restricting abortion access, rates of pregnancy-related kidney disease will likely continue to increase.^17,18^

Our findings highlight significant concerns regarding the long-term care of individuals who experienced pregnancy-related ESKD, as we found an alarming lack of access to kidney transplant and predialysis care, consistent with prior studies documenting stark racial disparities in access.^32,33,34,35^Those with pregnancy-related ESKD were significantly less likely to have nephrology care or be informed about kidney transplant before ESKD onset, and only 4% had a graft or fistula placed. Although it is possible this reflects the need for rapid dialysis initiation in the setting of an acute obstetric event such as massive hemorrhage or maternal septic shock, it also serves as a potential area for improvement in care. Outside of pregnancy, early access to nephrology care has been repeatedly shown to improve dialysis outcomes and likelihood of receiving a kidney transplant.^36,37,38^ Importantly, we also found that the disparity in access to transplant was attenuated among individuals with pregnancy-related ESKD who had nephrology care or a graft or fistula placed before onset. These findings underscore the critical importance of antepartum recognition of AKI, early referral to nephrology, and long-term follow-up with appropriate specialists.

Regarding the mechanisms underlying the observed disparities in access to transplant, we suspected that patients with pregnancy-related ESKD might be less ideal candidates for kidney transplant due to a higher burden of underlying comorbidities or worse functional status. However, this is not supported by our findings, as patients with pregnancy-related ESKD were younger and had lower rates of comorbidities compared with those with other causes of ESKD. We furthermore speculated that increased sensitization (eg, the presence of circulating antibodies to potential donor antigens), either from pregnancy itself or from blood transfusion in the setting of obstetric hemorrhage, might result in lower transplant rates.^39^ However, our findings also do not support this hypothesis, as waiting time once listed was similar, not increased, as would be expected if higher sensitization was a major contributor to decreased transplant rates. Given that the disparity in access was primarily observed in joining the transplant waitlist, the primary reasons may be more related to social determinants of health including inadequate insurance coverage, lack of financial resources, language barriers, transportation difficulties, as well as structural, institutional, and systemic racism. Notably there are known racial disparities in both joining the kidney transplant waitlist and receiving a live donor transplant for Black individuals that may not be entirely explained by social determinants of health.^32,40,41,42^ Maternal factors including having to care for a newborn may also contribute to reduced access, particularly in light of the high risk of preterm birth and prolonged neonatal intensive care unit admission among infants born to women with AKI.^4,5,6^ More work is needed to better understand these potential contributing factors to facilitate interventions to improve care.

We found that Black individuals were at increased risk of pregnancy-related ESKD, comprising 31.9% of these patients compared with only 16.2% of the general pregnant population over the same period. Racial disparities in both pregnancy and ESKD outcomes are well documented.^7,8,10,43,44^ Prior studies have shown a 2-fold increase in the rate of preterm birth and twice the risk of infant mortality for Black patients compared with non-Hispanic White patients.^45,46,47^ Among those with ESKD, Black patients under the age of 50 have up to twice the risk of death while on dialysis and are significantly less likely to receive a kidney transplant.^43^ Unfortunately, our paper adds to the evidence of racial disparities placing Black individuals at higher risk for adverse pregnancy outcomes, including pregnancy-related ESKD.

### Limitations

Our study has several limitations that merit consideration. Only the most extreme cases of pregnancy-related ESKD in which the nephrologist felt that pregnancy was a primary vs a contributing cause were captured in our study, likely underestimating the true burden of ESKD related to pregnancy. The use of the 2728 form also introduces the potential for misclassification in primary cause of ESKD as previously described in the literature.^48,49^ We suspect patients are less likely to be incorrectly classified as having pregnancy-related ESKD—likely given the typically close temporal relationship between the pregnancy and ESKD diagnosis for this to be considered a primary cause. Prior work suggests Black patients may have a higher likelihood of being incorrectly categorized as having hypertension as their primary cause.^48,50,51^ If Black patients with a pregnancy-related cause were more likely to be incorrectly classified as having a hypertensive primary cause, our results may underestimate the true racial disparity in pregnancy-related ESKD. Likewise, given the nature of the data, we lacked details on the specific clinical events that led to a diagnosis of pregnancy-related ESKD. Data are derived from form 2728, which is signed by the filing clinician and patient, but the process for ascertainment of information and review for accuracy may vary by center or individual practitioner. Additionally, comorbidities are reported on the medical evidence form at the time of ESKD onset, thus we were unable to account for changes in comorbidity status or severity over time, which may have negatively impacted access to transplant.

## Conclusions

Using the USRDS national registry, our findings highlight the racial disparities in incidence of pregnancy-related ESKD and need for improvement in long-term care of this vulnerable population. Despite having equivalent or better survival with ESKD, access to transplant among those with pregnancy-related ESKD was surprisingly low, and appeared to be at least partly related to a lack of pre-ESKD nephrology care. Though our study only included those with a pregnancy-related primary cause of ESKD, our findings may serve as a foundation to guide clinical care and future research within the much larger population of patients with pregnancy-related AKI who are at significant risk for mortality and severe morbidity.

## Notes

Supplement 1. **eTable 1.** Multivariable Cox Proportional Hazards Model: Time to Death Among Reproductive Age Females With End-Stage Kidney Disease **eTable 2.** Multivariable Cox Proportional Hazards Model: Time to Death Among Reproductive Age Females With End-Stage Kidney Disease in Cohort With Pregnancy-Related End-Stage Kidney Disease Exact Matched on Age to Patients With Other Causes of End-Stage Kidney Disease **eTable 3.** Multivariable Competing Risk Model: Access to Transplant Among Reproductive Age Females With End-Stage Kidney Disease, Treating Death as a Competing Risk **eTable 4.** Adjusted Hazard of Access to Transplant for Patients With Pregnancy-Related End Stage Kidney Disease vs Other Causes, From Multivariable Competing Risk Models, Stratified by Presence or Absence of Pre–End Stage Kidney Disease Care

Supplement 2.
Data Sharing Statement
