# Supplementary material for: Characteristics and Outcomes of Patients With Pregnancy-Related End-Stage Kidney Disease
Source: JAMA Netw Open. 2023 Dec 8;6(12):e2346314. doi: 10.1001/jamanetworkopen.2023.46314 (PMC10709776; doi:10.1001/jamanetworkopen.2023.46314)
Supplement: Supplement 1. — eTable 1. Multivariable Cox Proportional Hazards Model: Time to Death Among Reproductive Age Females With End-Stage Kidney Disease eTable 2. Multivariable Cox Proportional Hazards Model: Time to Death Among Reproductive Age Females With End-Stage Kidney Disease in Cohort With Pregnancy-Related End-Stage Kidney Disease Exact Matched on Age to Patients With Other Causes of End-Stage Kidney Disease eTable 3. Multivariable Competing Risk Model: Access to Transplant Among Reproductive Age Females With End-Stage Kidney Disease, Treating Death as a Competing Risk eTable 4. Adjusted Hazard of Access to Transplant for Patients With Pregnancy-Related End Stage Kidney Disease vs Other Causes, From Multivariable Competing Risk Models, Stratified by Presence or Absence of Pre–End Stage Kidney Disease Care [file jamanetwopen-e2346314-s001.pdf]

Supplemental Online Content

Kucirka LM, Angarita AM, Manuck TA, et al. Characteristics and outcomes of patients with pregnancy-related end-stage kidney disease. *JAMA Netw Open*. 2023;6(12):e2346314. doi:10.1001/jamanetworkopen.2023.46314

eTable 1. Multivariable Cox Proportional Hazards Model: Time to Death Among Reproductive Age Females With End-Stage Kidney Disease

eTable 2. Multivariable Cox Proportional Hazards Model: Time to Death Among Reproductive Age Females With End-Stage Kidney Disease in Cohort With Pregnancy-Related End-Stage Kidney Disease Exact Matched on Age to Patients With Other Causes of End-Stage Kidney Disease

eTable 3. Multivariable Competing Risk Model: Access to Transplant Among Reproductive Age Females With End-Stage Kidney Disease, Treating Death as a Competing Risk

eTable 4. Adjusted Hazard of Access to Transplant for Patients With Pregnancy-Related End Stage Kidney Disease vs Other Causes, From Multivariable Competing Risk Models, Stratified by Presence or Absence of Pre–End Stage Kidney Disease Care

This supplemental material has been provided by the authors to give readers additional information about their work.

© 2023 Kucirka LM et al. *JAMA Network Open.*

eTable 1. Multivariable Cox Proportional Hazards Model: Time to Death Among Reproductive Age Females With End-Stage Kidney Disease

Covariate	HR (95% CI)	p-value
Age (years)	1.03 (1.03-1.03)	<0.001
Primary cause of kidney failure*		
Pregnancy-related vs	1.00	
Glomerulonephritis/cystic kidney	0.96 (0.76-1.19)	0.7
Diabetes/hypertension	0.49 (0.39-0.61)	<0.001
Other/unknown	0.60 (0.48-0.75)	<0.001
Race		
White	1.00	
Black	0.95 (0.94-0.97)	<0.001
Asian/Pacific Islander	0.61 (0.58-0.63)	<0.001
Other/unknown	1.04 (1.00-1.09)	0.04
Hispanic Ethnicity	0.73 (0.71-0.74)	<0.001
Insurance type		
Private insurance	1.00	
Medicaid only	1.74 (1.71-1.78)	<0.001
Medicare/Medicare Advantage only	1.80 (1.74-1.85)	<0.001
Medicaid and Medicare	2.04 (1.99-2.10)	<0.001
DVA or other	1.27 (1.23-1.31)	<0.001
None	1.38 (1.35-1.41)	<0.001
Currently Employed	0.87 (0.86-0.89)	<0.001
Congestive heart failure	1.47 (1.44-1.49)	<0.001
Chronic obstructive pulmonary disease	1.21 (1.17-1.26)	<0.001
Cerebrovascular disease	1.16 (1.12-1.19)	<0.001
Alcohol dependence	1.34 (1.27-1.41)	<0.001
Hypertension	0.86 (0.71-0.83)	<0.001
Inability to ambulate	1.77 (1.71-1.82)	<0.001
Current smoker	1.32 (1.29-1.36)	<0.001

Inverse hazard ratios shown for cause of renal failure, so should be interpreted as the hazard for patients with pregnancy-related ESKD compared to each of the other causes Model adjusted for potential confounders including race, Hispanic Ethnicity, insurance type, current employment, and comorbidities including congestive heart failure, chronic obstructive pulmonary disease, cerebrovascular disease, alcohol dependence, hypertension, inability to ambulate, current smokerDVA = Department of Veterans' Affairs*

© 2023 Kucirka LM et al. *JAMA Network Open.*

eTable 2. Multivariable Cox Proportional Hazards Model: Time to Death Among Reproductive Age Females With End-Stage Kidney Disease (ESKD) in Cohort With Pregnancy-Related ESKD Exact Matched on Age to Patients With Other Causes of ESKD

**Covariate**	**HR (95% CI)**	**p-value**
Age (years)	1.03 (1.02-1.04)	<0.001
Primary cause of kidney failure*		
Pregnancy-related vs	1.00	
Glomerulonephritis/cystic kidney	1.06 (0.79-1.41)	0.7
Diabetes/hypertension	0.56 (0.43-0.74)	<0.001
Other/unknown	0.67 (0.49-0.91)	0.01
Race		
White	1.00	
Black	1.15 (0.95-1.39)	0.1
Asian/Pacific Islander	0.91 (0.59-1.38)	0.6
Other/unknown	0.72 (0.41-1.23)	0.2
Hispanic Ethnicity	0.86 (0.67-1.09)	0.2
Insurance type		
Private insurance	1.00	
Medicaid only	1.80 (1.43-2.27)	<0.001
Medicare/Medicare Advantage only	2.01 (1.36-2.99)	<0.001
Medicaid and Medicare	2.26 (1.64-3.12)	<0.001
DVA or other	1.21 (0.89-1.66)	0.3
None	1.23 (0.92-1.64)	0.2
Currently Employed	0.89 (0.75-1.06)	0.2
Congestive heart failure	1.58 (1.27-1.97)	<0.001
Chronic obstructive pulmonary disease	1.23 (0.75-1.00)	0.4
Cerebrovascular disease	1.30 (0.86-1.96)	0.6
Alcohol dependence	1.76 (0.71-4.31)	0.2
Hypertension	0.98 (0.77-1.24)	0.9
Inability to ambulate	1.35 (0.80-2.25)	0.3
Current smoker	1.61 (1.23-2.10)	<0.001

Inverse hazard ratios shown for cause of renal failure, so should be interpreted as the hazard for patients with pregnancy-related ESKD compared to each of the other causes Model adjusted for potential confounders including race, Hispanic Ethnicity, insurance type, current employment, and comorbidities including congestive heart failure, chronic obstructive pulmonary disease,cerebrovascular disease, alcohol dependence, hypertension, inability to ambulate, current smoker*
DVA = Department of Veterans' Affairs

© 2023 Kucirka LM et al. *JAMA Network Open.*

eTable 3. Multivariable Competing Risk Model: Access to Transplant* Among Reproductive Age Females With End-Stage Kidney Disease, Treating Death as a Competing Risk

	Access to transplant*		Time to transplant after listing	
**Covariate**	**aSHR (95% CI)**	**p-value**	**aSHR (95% CI)**	**p-value**
Age (years)	0.97 (0.97-0.97)	<0.001	0.98 (0.98-0.98)	<0.001
Primary cause of kidney failure**				
Pregnancy-related vs	1.00		1.00	
Glomerulonephritis/cystic kidney	0.51 (0.43-0.63)	<0.001	0.91 (0.72-1.16)	0.4
Diabetes/hypertension	0.81 (0.67-0.98)	0.02	1.23 (0.95-1.54)	0.1
Other/Unknown	0.82 (0.67-0.99)	0.03	0.92 (0.71-1.16)	0.4
Race				
White	1.00		1.00	
Black	0.75 (0.73-0.76)	<0.001	0.57 (0.55-0.59)	<0.001
Asian/Pacific Islander	1.14 (1.11-1.18)	<0.001	0.69 (0.67-0.72)	<0.001
Other/unknown	0.80 (0.76-0.84)	<0.001	0.78 (0.73-0.84)	<0.001
Hispanic Ethnicity	0.99 (0.97-1.01)	0.4	0.72 (0.69-0.74)	<0.001
Insurance type				
Private insurance	1.00		1.00	
Medicaid only	0.45 (0.44-0.46)	<0.001	0.63 (0.61-0.64)	<0.001
Medicare/Medicare Advantage only	0.51 (0.49-0.53)	<0.001	0.68 (0.65-0.72)	0.5
Medicaid and Medicare	0.43 (0.41-0.44)	<0.001	0.60 (0.57-0.64)	<0.001
DVA or other	0.73 (0.71-0.75)	<0.001	0.88 (0.85-0.91)	<0.001
None	0.49 (0.48-0.51)	<0.001	0.75 (0.73-0.78)	<0.001
Currently Employed	1.03 (1.01-1.05)	0.001	0.99 (0.96-1.01)	0.4
Congestive heart failure	0.61 (0.59-0.63)	<0.001	0.73 (0.69-0.75)	<0.001
Chronic obstructive pulmonary disease	0.51 (0.47-0.55)	<0.001	0.79 (0.70-0.90)	0.001
Cerebrovascular disease	0.741(0.67-0.75)	<0.001	0.89 (0.83-0.96)	0.001
Alcohol dependence	0.51 (0.45-0.57)	<0.001	1.11 (0.93-1.31)	0.2
Hypertension	1.19 (1.16-1.23)	<0.001	0.91 (0.89-0.94)	<0.001
Inability to ambulate	0.27 (0.25-0.29)	<0.001	0.81 (0.71-0.94)	0.005
Current smoker	0.58 (0.55-0.60)	<0.001	0.76 (0.72-0.81)	<0.001

Access to transplant defined as joining the deceased donor waitlist or receiving a kidney transplant from a live donor Inverse hazard ratios and sub-hazard ratios shown for cause of renal failure, so should be interpreted as the hazard for patients with pregnancy-related ESKD compared to each of the other causes*
aSHR = adjusted sub-hazard ratio
DVA= department of Veterans' Affairs

© 2023 Kucirka LM et al. *JAMA Network Open.*

eTable 4. Adjusted Hazard of Access to Transplant for Patients With Pregnancy-Related End Stage Kidney Disease vs Other Causes, From Multivariable Competing Risk Models, Stratified by Presence or Absence of Pre–End Stage Kidney Disease Care

	**Pre-End Stage Kidney Disease Care Measures**		
Pre-ESKD Care Measures	Early access to nephrology care	Graft or arteriovenous fistula at ESRD onset	Patient informed about option of transplant
	aHR* access to transplant for pregnancy-related ESKD vs other causes (95% CI)	aHR* access to transplant for pregnancy-related ESKD vs other causes (95% CI)	aHR* access to transplant for pregnancy-related ESKD vs other causes (95% CI)
Measure absent	**0.62 (0.45-0.83)**	**0.64 (0.50-0.81)**	0.59 (0.30-0-1.15)
Measure present	0.84 (0.62-1.14)	1.10 (0.55-2.19)	**0.67 (0.53-0.83)**

***BOLD** indicates p <0.05*

**aHR = adjusted hazard ratio Each model adjusted for age, race, ethnicity, insurance type, currently employed, and the presence or absence of comorbidities including congestive heart failure, chronic obstructive pulmonary disease, cerebrovascular disease, hypertension, alcohol abuse, current smoker, and inability to ambulate*

ESKD = end-stage kidney disease

© 2023 Kucirka LM et al. *JAMA Network Open.*
