# Supplementary material for: Characteristics and Outcomes of Patients With Pregnancy-Related End-Stage Kidney Disease
Source: JAMA Netw Open. 2023 Dec 8;6(12):e2346314. doi: 10.1001/jamanetworkopen.2023.46314 (PMC10709776; doi:10.1001/jamanetworkopen.2023.46314)
Supplement: Supplement 2. — Data Sharing Statement [file jamanetwopen-e2346314-s002.pdf]

Data Sharing Statement

Kucirka. Characteristics and Outcomes of Patients with Pregnancy-Related End-Stage Kidney Disease. *JAMA Netw Open*. Published December 08, 2023.
doi:10.1001/jamanetworkopen.2023.46314

Data
Data available: No

Additional Information
Explanation for why data not available: Not allowed under our data use agreement with the United States Renal Data System
